# Association of Firearm Ownership, Use, Accessibility, and Storage Practices With Suicide Risk Among US Army Soldiers

**DOI:** 10.1001/jamanetworkopen.2019.5383

**Published:** 2019-06-07

**Authors:** Catherine L. Dempsey, David M. Benedek, Kelly L. Zuromski, Charlotte Riggs-Donovan, Tsz Hin H. Ng, Matthew K. Nock, Ronald C. Kessler, Robert J. Ursano

**Affiliations:** 1 Center for the Study of Traumatic Stress, Department of Psychiatry, Uniformed Services University of the Health Sciences, Bethesda, Maryland; 2 Department of Psychology, Harvard University, Cambridge, Massachusetts; 3 Department of Healthcare Policy, Harvard Medical School, Boston, Massachusetts

## Abstract

**Importance:**

Since 2004, the suicide rate among US Army soldiers has exceeded the rate of death from combat injury. It is critical to establish factors that increase the risk of acting on suicidal thoughts to guide early intervention and suicide prevention.

**Objective:**

To assess whether firearm ownership, use, storage practices, and accessibility are associated with increased risk of suicide.

**Design, Setting, and Participants:**

In this case-control study, suicide cases (n = 135) were defined as US Army soldiers who died by suicide while on active duty between August 1, 2011, and November 1, 2013. Next-of-kin and Army supervisors of soldiers who died by suicide (n = 168) were compared with propensity-matched controls (n = 137); those soldiers with a suicidal ideation in the past year (n = 118) provided structured interview data. Data were analyzed from April 5, 2018, to April 2, 2019.

**Main Outcomes and Measures:**

Firearm ownership, storage, and accessibility were assessed by using items from the World Health Organization Composite International Diagnostic Interview screening scales along with items created for the purpose of the Army Study to Assess Risk and Resilience in Servicemembers (Army STARRS) study.

**Results:**

Among the 135 suicide decedents, next-of-kin reported that they had greater accessibility to firearms compared with propensity-matched controls. Specifically, suicide decedents were more likely to own 1 or more handguns compared with propensity-matched controls (odds ratio [OR], 1.9; 95% CI, 1.0-3.7; χ^2^_1_ = 4.2; false discovery rate [FDR] *P* = .08), store a loaded gun at home (OR, 4.1; 95% CI, 1.9-9.1; χ^2^_1_ = 12.2; FDR *P* = .003), and publicly carry a gun when not required for military duty (OR, 3.2; 95% CI, 1.4-7.3; χ^2^_1_ = 7.4; FDR *P* = .02). The combination of these 3 items was associated with a 3-fold increase in the odds of suicide death (OR, 3.4; 95% CI, 1.2-9.4; χ^2^_1_ = 5.4; FDR *P* = .05). Storing a loaded gun with ammunition at home or publicly carrying a gun when not on duty was associated with a 4-fold increase in the odds of suicide death (OR, 3.9; 95% CI, 1.9-7.9; χ^2^_1_ = 14.1; FDR *P* = .002).

**Conclusions and Relevance:**

In this study, in addition to gun ownership, ease and immediacy of firearm access were associated with increased suicide risk. Discussion with family members and supervisors about limiting firearm accessibility should be evaluated for potential intervention.

## Key Points

**Question:**

To what extent do firearm ownership, use, accessibility, and storage practices increase suicide risk among US Army soldiers?

**Findings:**

In this case-control, psychological autopsy study of 135 soldiers who died by suicide, firearm accessibility was associated with a significant increase in the risk of suicide. Next-of-kin reported that soldiers who died by suicide were more likely to own firearms, have home access to firearms, and have home storage of ammunition compared with propensity-matched controls.

**Meaning:**

This study suggests that identifying possible targets for intervention, such as promoting the separate storage of guns and ammunition as well as discouraging public carrying of firearms when not required for military duties, has important implications for suicide prevention.

## Teaser

This case-control study uses data from the Soldier Health Outcomes Study conducted as part of the Army Study to Assess Risk and Resilience in Servicemembers (Army STARRS) to evaluate whether increased accessibility to firearms is associated with an increased risk of suicide among US Army soldiers.

## Introduction

The suicide rate among soldiers in the US Army increased substantially during the Afghanistan-Iraq wars, peaked in 2012, and continues to exceed the rate of combat deaths.^1,2^ The rise in suicides among US Army soldiers reflects a national issue that extends beyond the military population.^3^ Multiple studies^4,5^ have shown an association between firearm ownership and suicide in the general population. Since many military service personnel own firearms, keep them in their homes, carry them, and use them regularly, we sought to assess whether increased accessibility to firearms is associated with increased risk of suicide.

Among methods used during suicides, firearms are associated with the highest rates of suicide mortality. Hilton et al^6^ studied Navy suicides between 1999 and 2007 and reported that accessibility to firearms appeared to influence method selection, given that 69% of decedents with access to military firearms used such a weapon in their fatal suicide attempt compared with 52% of decedents without such access. The authors also suggested the importance of firearm training, and they found that 69% of individuals who had received military weapons training used a firearm compared with 49% of those without such training.

However, individual-level studies of suicidal decedents based on psychological autopsy methodologies provided strong evidence of associations between firearm access and suicide. It has been reported, for example, that both males and females who died by suicide were more likely to have died in a home containing a firearm, independent of whether they lived alone or with others.^7^ In another study, firearm possession was positively associated with firearm suicide and was inversely associated with nonfirearm suicide.^8^ In some studies with similar outcomes, analyses were further adjusted for demographic, socioeconomic, and clinical variables.^9,10^

In addition to ownership of firearms, several individual-level studies have indicated that unsafe storage of firearms was associated with an increased risk of suicide. In one such study,^11^ storing the firearm loaded and unlocked was an independent predictor of suicide. In another report,^12^ guns in households of the case group were less likely to be stored unloaded compared with guns in the control group. Similarly, guns in the case group were less likely to be stored locked, to be stored separately from ammunition, or to have ammunition that was locked compared with guns in the control group.

Other behaviors may be significant in relation to firearm accessibility and suicide risk. Two studies^13,14^ have documented positive associations between past suicide attempts and carrying a firearm within the previous 30 days. A third study^15^ reported a similar positive association between past suicide attempts and carrying a weapon in the previous 30 days, although in that case the weapon concerned was not necessarily a firearm.^15,16^

There is also evidence suggesting that greater experience and aptitude in the use of a firearm may confer a greater risk of suicide attempts.^16^ We note that, although existing work suggests an association between both suicide attempts and death and firearms, longitudinal and cross-sectional studies have thus far failed to identify clear associations between thinking about suicide and firearm access.^17,18^ Thus, it is likely that the association between firearms and suicide risk may be related to access, availability, and familiarity with a highly lethal method. Firearm experience is associated with lifetime suicide attempts, but not with suicidal ideation. Thus, a range of factors may confer risk of suicide among military personnel, namely, firearm ownership, accessibility of firearms, firearm storage practices, and off-duty firearm usage.

In this study, we report data from a psychological autopsy study, the Soldier Health Outcomes Study (SHOS-B), which was conducted as part of the Army Study to Assess Risk and Resilience in Servicemembers (Army STARRS).^19^ Our case-control study was designed to test whether the increased accessibility to firearms is associated with an increased risk of suicide. To our knowledge, this is the only study of its type relating to Army personnel on active service duties.

## Methods

### Sample

Suicide cases were US Army soldiers (n = 135) who died by suicide while on active duty between August 1, 2011, and November 1, 2013. This sample excluded soldiers in the Army Reserve and National Guard, soldiers who died by suicide while deployed, and soldiers outside of the continental United States. Excluded cases (n = 155) did not differ from the included cases for demographic or Army variables. The research team interviewed a next-of-kin and/or first-line Army supervisor of 135 soldiers among 290 eligible soldiers (46.6%) who died by suicide during the study period. Recruitment, informed consent, and data collection procedures, described in more detail elsewhere,^20^ were approved by the Humans Subjects Committees of the University of Michigan, Ann Arbor; the Uniformed Services University of the Health Sciences, Bethesda, Maryland; and all other collaborating organizations. Informed consent was obtained from the next-of-kin and supervisors of suicide cases. Controls also completed a SHOS-B consent and a consent to participate in the All ARMY Study (AAS), which included the consent to have their survey data linked. The study followed the Strengthening the Reporting of Observational Studies in Epidemiology (STROBE) reporting guideline. Statistical analysis was performed from April 5, 2018, to April 2, 2019.

Controls consisted of soldiers matched according to known sociodemographic and Army history risk factors for suicide death using propensity-score matching (n = 137) and soldiers with suicidal ideation in the past year (n = 118). Of the 137 propensity-matched controls, 128 had a next-of-kin responder and 80 had a supervisor responder (71 had both). Of the 118 controls with a history of suicidal ideation, 108 had a next-of-kin responder and 73 had a supervisor responder (63 had both).

In the control group, the first set of controls was drawn from participants in the Army STARRS AAS,^19^ a large (N = 5428) representative sample of soldiers. The propensity score used for sampling was created by estimating completed suicide by using multiple risk factors. These controls were matched to Army suicide decedents from 2004 through 2009 for the following variables: calendar month and year, person record, sex, age, race/ethnicity, marital status, number of dependents, educational level, rank, age at Army entry, deployment status (never or previously deployed), months since last deployment, episodes of continuous service, count of active-duty (full-time) months, count of total months in the Army, religion, number of times demoted, months since last demotion, number of times promoted, months since last promotion, Armed Forces Qualification Test score, current or previous stop-loss (involuntary extension of active duty), and number of previous injuries.

The second set of controls was selected from the AAS and had endorsed suicidal ideation in the past year per self-report (n = 118). This second comparison group was used to increase the ability to identify factors of suicide death beyond those for suicidal ideation. Controls were selected with replacement (no duplicate participants were present in the final analytic control samples) from both samples (propensity score method and 12-month ideators). Neither group of controls differed from eligible AAS respondents who did not participate on factors of sex, race/ethnicity, marital status, or age at entry into the Army.

### Weighting Procedures

Cases were weighted to all suicide deaths recorded in the Armed Forces Medical Examiner Tracking System between August 1, 2011, and October 31, 2013, that were Regular Army, continental United States. The Armed Forces Medical Examiner Tracking System includes manner of death and cause of death, including those self-inflicted. Controls with suicidal ideation in the past year were weighted to the US Army population (with suicidal ideation in the past year) via the AAS population. Similarly, the propensity-matched controls without suicidal ideation were separately weighted to the Army population (without suicidal ideation) via the AAS population.

Poststratification weights were developed based on the analysis of the Historical Administrative Data Study Army sample^21^ by using factors associated with suicide found in administrative records and known population information gathered from the Army snapshot data set (a monthly picture of demographic information of all Army soldiers). The Historical Administrative Data Study is an integrated administrative data file containing key elements from 38 different Army and Department of Defense data systems for more than 1.6 million soldiers (regular Army, Army Reserve, and National Guard) on active duty during calendar years 2004 through 2009. Cases were adjusted to match the population of all deaths in the Army, whereas controls were adjusted to match the AAS, a large and representative sample of active-duty soldiers.^22^ Because controls were selected using 2 different criteria (12-month ideation or propensity score), weights were separately calculated for method of selection. In total, 3 separate weights were created using the same method. The steps involved in creating poststratification weights included: (1) using demographic and Army-related variables in a forward stepwise regression model to choose important variables associated with participation in the study, (2) modifying weights to reflect the population distribution on the regression variables, (3) trimming large weights, and (4) normalizing the weights to reflect original sample size counts.

### Measures

The SHOS-B interview assessed a broad range of potential risk and protective factors for suicide: history of mental health diagnoses, mental health services utilization, lifetime and recent stressful events, potential resilience factors, personality characteristics, previous history of suicidal thoughts and behavior, and informant perspectives.

We assessed firearm ownership and storage practices by using items from the World Health Organization Composite International Diagnostic Interview screening scales (CIDI-SC) as well as items created for the purpose of the Army STARRS study.^21,23,24^ Supervisors were asked the following questions about firearm use: “To the best of your knowledge, has the soldier ever aimed a gun at another person?” and “To the best of your knowledge, has the soldier ever fired a gun at another person?” Supervisors could respond to this question by answering yes, no, or do not know. The do not know responses were collapsed with the no responses to create dichotomized constructs for these questions.

In addition to the questions asked of the supervisors, the next-of-kin were asked more in-depth questions about firearm ownership, storage practices, use, and accessibility since they may have had better knowledge and proximity to the soldier’s personal firearm practices. Next-of-kin items included the following: (1) How many handguns in working condition did he/she have in his/her home? (2) How many rifles or shotguns in working condition did he/she have in his/her home? (3) How many guns in his/her home were stored in a safe or lock-box or fitted with a safety lock? (4) How many guns in his/her home were stored loaded with ammunition? (5) Has the soldier ever aimed a gun at another person? (6) Has the Soldier ever fired a gun at another person? (7) Not counting times he/she was on duty, how often did he/she carry a gun with him/her (or in his/her vehicle) when he/she was out in his/her neighborhood (eg, going for a walk or to the grocery store)? and (8) Not counting times he/she was on duty, how often did he/she carry some other weapon such as a knife, club, or mace with him/her when he/she was/is out in his/her neighborhood (eg, going for a walk or to the grocery store)? Responses to question 2 were collapsed to form 3 analytic categories (0 rifles or shotguns, 1 rifle or shotgun, and ≥2 rifles or shotguns). Responses from question 1 (number of working handguns in the home) and question 2 (number of working rifles or shotguns in the home) were added to calculate the total number of working firearms in the home. After adding the responses from the 2 questions, the variable was dichotomized, resulting in the following analytic categories: 0 firearms and 1 or more firearms. This total firearm item was then combined with questions 4 and 7 to create the increased accessibility item: owns a gun, keeps firearms loaded in the home, and carries firearms around the neighborhood. Question 8 has 3 analytic categories: none of the time, some of the time, and most of the time. The other responses to the questions in the next-of-kin section were dichotomized.

### Statistical Analysis

We compared cases and controls for sociodemographic and Army history variables using Wald χ^2^ tests. Variables that emerged as significantly different (for next-of-kin: deployment status and number of years of active service; for supervisor: deployment status) were retained as covariates in all subsequent analyses (eTable in the Supplement). Odds ratios (ORs) and 95% CIs were also estimated. The limits of the CI are rounded to the nearest tenth (ie, if the true value for this estimate is 1.01, it was rounded to 1.0). We used a series of multivariate logistic regression analyses to assess the outcome of suicide case status (no or yes) and potential risk factors (firearm use, accessibility, and storage practices). Coefficients were exponentiated in logistic models to create ORs with 95% CIs. To correct for multiple comparisons, we used the false discovery rate (FDR) within each sample (next-of-kin or supervisor) for (1) past years suicidal ideation and (2) propensity-matched comparisons. The FDR was conducted using the *p.adjust* function in R, version 3.4.2 (R Foundation). All other analyses were conducted using SAS, version 9.4 (SAS Institute Inc).

Item-level missing data were handled in a 3-step process described in the Army STARRS study design and methods publication.^21^ Linkage to administrative data was used for missing demographics and Army characteristic variables; if data were not available from the administrative linkage, they were listed as missing and not included in the analyses. For variables based on survey items, responses were imputed to the null analytic category if they could be defined as a nonendorsed, nonmissing response. This type of response can be defined as being 1 of the following: do not know, refusal, skip (because of the skip pattern logic prescribed by the survey structure), or not applicable. If the constructed survey variable lacked a null analytic category, then these nonendorsed, nonmissing responses were imputed to the mode response. To assess the significance of the association between the independent variables and the outcome variable, omnibus χ^2^ tests were performed when fitting each of the logistic regression models. If *P* < .05 for the resulting χ^2^ for a given omnibus test, the independent variable was considered to be significantly associated with the outcome of suicide death. All tests were 2-sided.

## Results

Of the 135 soldiers who died by suicide, 111 (82.2%) had a documented method of injury associated with their suicide death in a Department of Defense Suicide Event Report,^2^ which standardizes suicide surveillance efforts across the military services and tracks the total suicide deaths, manner of death, and other variables. Firearms were the most common method of injury identified with 61 of the 111 soldiers (55.0%) dying by this method. Among those who died by firearm suicide, 58 (95.1%) used a firearm that was not a military issue or duty weapon. Of these 61 firearm suicides, 46 (75.4%) were reported to have a gun in the home or immediate environment. After firearms, hanging or asphyxiation was the next most frequently reported method of injury, with 35 of the 111 soldiers (32.0%) dying by this method.

Next-of-kin reported that suicide decedents had greater firearm access than propensity-matched controls. (Table 1). Specifically, suicide decedents were more likely to own 1 or more handguns compared with propensity-matched controls (OR, 1.9; 95% CI, 1.0-3.7; χ^2^_1_ = 4.2; FDR *P* = .08), store a gun loaded with ammunition at home (OR, 4.1; 95% CI, 1.9-9.1; χ^2^_1_ = 12.4; FDR *P* = .003), and carry a personal gun in public (OR, 3.2; 95% CI, 1.4-7.3; χ^2^_1_ = 7.8; FDR *P* = .02). The combination of these 3 items was associated with a 3-fold increase in the odds of suicide (OR, 3.4; 95% CI, 1.2-9.4; χ^2^_1_ = 5.8; FDR *P* = .05). The increased accessibility of either storing a loaded gun with ammunition at home or carrying a personal gun in public resulted in a 4-fold increase of the odds of suicide (OR, 3.9; 95% CI, 1.9-7.9; χ^2^_1_ = 14.1; FDR *P* = .002). Of interest, next-of-kin reported that use of safety locks at home was protective for suicide decedents compared with propensity-matched controls, but this difference was not significant (OR, 0.9; 95% CI, 0.4-1.7; χ^2^_1_ = 0.2; FDR *P* = .78). The US Army supervisors of suicide decedents did not observe significant differences between cases and controls (Table 2).

**Table 1.** Firearm Accessibility Reported by Next-of-Kin

Characteristic	Cases, No. (%) (n = 61)	Controls					
		12-mo Suicidal Ideation (n = 108)			Propensity Score–Matched (n = 128)		
		No. (%)	OR (95% CI)^a^^,^^b^	FDR *P* Value^c^	No. (%)	OR (95% CI)^a^^,^^b^	FDR *P* Value^c^
≥1 Working handgun in home	30 (52)	37 (33)	2.1 (0.6-8.1)	.70	45 (40)	1.9 (1.0-3.7)	.08
≥1 Working rifle or shotgun in home	16 (26)	30 (28)	0.9 (0.2-3.6)	.94	32 (25)	1.5 (0.7-2.9)	.40
≥1 Handgun, rifle, or shotgun owned	32 (55)	42 (40)	1.8 (0.5-6.5)	.70	51 (43)	1.9 (1.0-3.6)	.08
≥1 Gun loaded, and ammunition stored at home	15 (26)	8 (7)	4.5 (0.4-45.4)	.70	11 (9)	4.1 (1.9-9.1)	.003
Guns at home fitted with safety locks	16 (26)	36 (33)	0.8 (0.2-3.2)	.94	41 (32)	0.9 (0.4-1.7)	.78
Aimed a gun at another person	14 (24)	29 (25)	0.8 (0.2-4.0)	.94	38 (25)	1.1 (0.5-2.2)	.94
Fired a gun at another person	15 (26)	27 (24)	1.1 (0.2-5.1)	.94	38 (28)	1.0 (0.5-2.0)	.95
Carried gun when in neighborhood	12 (21)	5 (4)	5.3 (0.3-93.2)	.70	9 (8)	3.2 (1.4-7.3)	.02
How often did/does he or she carry another weapon when out in the neighborhood (not including duty)							
Most of the time vs none	12 (23)	16 (16)	1.7 (0.3-9.8)	.94	19 (15)	2.2 (1.0-4.7)	.23
Some of the time vs none	4 (9)	11 (9)	0.9 (0.1-8.1)		8 (6)	1.3 (0.4-4.1)	
Owns a firearm, keeps it loaded, and carries it around the neighborhood	7 (13)	2 (2)	8.6 (0.1-803.5)	.70	4 (5)	3.4 (1.2-9.4)	.05
Keeps a loaded firearm or carries a firearm around the neighborhood	20 (33)	11 (10)	4.3 (0.6-37.7)	.70	16 (12)	3.9 (1.9-7.9)	.002

Abbreviations: FDR, false discovery rate; OR, odds ratio.

^a^ The OR statistics were obtained from separate multivariate logistic regression models testing differences between cases and each control group.

^b^ Each factor was adjusted for deployment status (never vs previously) and the number of years of active service (1-4, 5-8, or ≥9 years), but not for each other.

^c^ *P* values have been corrected using FDR. *P* values were corrected among next-of-kin for 12-month ideator and propensity score–matched comparisons.

**Table 2.** Firearm Accessibility Reported by Supervisors

Characteristic	Cases, No. (%) (n = 107)	Controls					
		12-mo Suicidal Ideation (n = 73)			Propensity Score–Matched (n = 80)		
		No. (%)	OR (95% CI)^a^^,^^b^	FDR *P* Value^c^	No. (%)	OR (95% CI)^a^^,^^b^	FDR *P* Value^c^
Aimed a gun at another person	19 (16)	17 (20)	0.8 (0.1-5.4)	.95	19 (24)	0.7 (0.4-1.4)	.66
Fired a gun at another person	15 (13)	13 (14)	0.9 (0.1-7.8)	.95	16 (17)	0.8 (0.4-1.8)	.67

Abbreviations: FDR, false discovery rate; OR, odds ratio.

^a^ OR statistics obtained from separate multivariate logistic regression models testing differences between cases and each control group.

^b^ Each predictor was adjusted for deployment status (never or previously), but not for each other.

^c^ *P* values have been corrected using FDR. *P* values were corrected among supervisor for 12-month ideator and propensity score–matched comparisons.

## Discussion

These results suggest that availability and access to firearms were significantly associated with suicide death among US soldiers on active duty. Our findings concurred with earlier studies^15,16^ by showing that factors beyond ownership of a firearm were associated with an increased risk of suicide. Specifically, suicide risk was associated with the ownership of 1 or more firearms, the storage of a firearm loaded with ammunition at home, and the carrying of a firearm in public while off-duty.

Recent theoretical work may help to explain our observed association between firearm access and suicide risk. Some current theories of suicide (eg, the interpersonal theory of suicide)^25^ suggest that fatal suicidal behavior results require not only the presence of suicidal desire but also a developed capability or capacity for suicidal behavior. According to the interpersonal theory of suicide, this capability for lethal self-injury is acquired through repeated exposure to painful and fear-inducing experiences, thus habituating an individual to the pain and fear required to enact a fatal suicide attempt.^26^ These experiences may include previous suicidal behavior, nonsuicidal self-harm, and physical abuse.^27^ Of note, exposure to the pain of other people may also induce the capacity for suicide.^28^ For soldiers on active duty, such experiences may be commonplace, and it is possible that our study has identified a subset of service personnel with an increased suicide capability and a reduced fear of death. Other earlier work^29^ found that military personnel who kept their firearms loaded and stored in unsecured locations exhibited higher mean levels of fearlessness about death. Future research may explore associations between increased capability of suicide and sensation seeking and stoicism.^30^

Our results also indicated that continued focus on *means restriction*^31^ (defined as limitation of access to lethal methods used for suicide, also known as *means safety*) counseling is warranted. Several widely used interventions already include a component on removing access to methods that would potentially be used in a suicide attempt (eg, safety planning).^32^ More recently, work on interventions primarily focused on means restriction has increased, including motivational interviewing for means restriction and lethal means counseling training for mental health professionals.^33^ We found that certain firearm-related variables were more robustly associated with suicide; thus, our results suggest areas for explicit focus within these existing interventions (eg, separate storage of guns and ammunition and limiting the carrying of firearms in public). Outside the realm of mental health research, focus on these types of firearm safety variables has been shown to significantly improve firearm storage practices.^28^

### Limitations

Several limitations must be considered when interpreting these findings. First, the relatively small sample size limited the ability to detect smaller effects or to test the interactions between identified risk and protective factors. Although several factors observed by next-of-kin distinguished suicide decedents from propensity-matched controls, none of these factors distinguished suicide decedents from the subset of controls who reported suicidal ideation within the past year. The supervisors were not familiar with the home firearm storage practices and were not asked the same questions. As a result, supervisor reports may have failed to estimate suicide risk because they did not have knowledge of the behavior of individual soldiers in relation to practices at home.

Our response rates were relatively low compared with surveys conducted in the general population, but they were high for multi-informant interviews conducted within a military population.^34,35^ Future research should focus on study design efforts to facilitate larger sample sizes. These efforts could include developments in outreach and recruitment efforts to contact family members who knew the decedent well but who are difficult to reach. Also, we lacked sufficient analytical capacity to conduct analyses by sex. Therefore, it was not possible to identify sex-specific associations for male and female soldiers who died by suicide.

## Conclusions

Our results suggest that, among US Army soldiers on active duty, availability and access to firearms may be associated with increased risk of suicide. These findings suggested that continued focus on lethal means counseling is warranted. Based on our results, specific targets within this intervention may include discussion and counseling associated with the separate storage of guns and ammunition and limiting the public carrying of firearms. In combination with data on other risk factors, many of which have been identified by other Army STARRS work,^36,37^ future research in this area may consider taking a precision psychiatry approach^38^ in which subsets of firearm-owning service personnel are identified who would particularly benefit from this type of intervention.

## Notes

Supplement. **eTable.** Comparison of Cases and Controls on Sociodemographic and Army History Variables
