# Supplementary material for: Association of Firearm Ownership, Use, Accessibility, and Storage Practices With Suicide Risk Among US Army Soldiers
Source: JAMA Netw Open. 2019 Jun 7;2(6):e195383. doi: 10.1001/jamanetworkopen.2019.5383 (PMC6563574; doi:10.1001/jamanetworkopen.2019.5383)
Supplement: Supplement. — eTable. Comparison of Cases and Controls on Sociodemographic and Army History Variables [file jamanetwopen-2-e195383-s001.pdf]

Supplementary Online Content

Dempsey CL, Benedek DM, Zuromski KL, et al. Association of firearm ownership, use, accessibility, and storage practices with suicide risk among US Army soldiers. *JAMA Netw Open*. 2019;2(6):e195383. doi:10.1001/jamanetworkopen.2019.5383

eTable. Comparison of Cases and Controls on Sociodemographic and Army History Variables

This supplementary material has been provided by the authors to give readers additional information about their work.

© 2019 Dempsey CL et al. *JAMA Network Open.*

eTable. Comparison of Cases and Controls on Sociodemographic and Army History Variables

Demographic Variables	Next of Kin: Cases (n = 61)	Next of Kin: Controls (Propensity) (n = 128)			Next of Kin: Controls (12-month ideation) (n = 108)			Supervisor: Cases (n = 107)	Supervisor: Controls (Propensity) (n = 80)			Supervisor: Controls (12-month ideation) (n = 73)		
	%	%	OR	(CI)	%	OR	(CI)	%	%	OR	(CI)	%	OR	(CI)
**Gender**														
Female	8.2	7.8	1.0	(0.4-3.0)	9.0	0.9	(0.1-7.8)	12.2	9.8	1.3	(0.6-2.9)	0.3	1.0	(0.1-8.8)
Male	91.8	92.2		-	91.0		-	87.9	90.3		-	87.7		-
**Marital Status**														
Never Married	34.6	28.9	1.4	(0.8-2.7)	30.2	1.3	(0.3-5.0)	34.8	32.2	1.2	(0.7-2.1)	32.2	1.1	(0.2-5.4)
Previously Married	10.2	4.3	2.8	(1.0-8.4)	7.3	1.6	(0.2-16.6)	9.2	5.1	2.0	(0.7-5.6)	10.6	0.9	(0.1-9.9)
Currently Married	55.3	66.8		-	62.5		-	56.1	62.7		-	57.2		-
**Race/ethnicity**														
White	71.1	63.3		-	69.1		-	65.1	60.2		-	64.3		-
Black	19.3	16.3	1.1	(0.5-2.2)	13.1	1.4	(0.2-8.7)	17.7	21.3	0.8	(0.4-1.5)	12.6	1.4	(0.2-12.6)
Hispanic	5.2	16.5	**0.3***	**(0.1-0.9)**	9.5	0.5	(0.1-5.1)	8.9	14.4	0.6	(0.2-1.3)	13.8	0.6	(0.1-5.5)
Asian /Other	4.4	3.9	1.0	(0.2-4.1)	8.4	0.5	(0.0-5.6)	8.4	4.2	1.9	(0.6-5.5)	9.3	0.9	(0.1-11.2)
**Rank**														
E1-E4 vs. E5-E9	48.8	61.0	**0.5***	**(0.3, 1.0)**	54.7	0.6	(0.2, 2.4)	51.2	48.9	1.0	(0.6, 1.7)	54.3	0.8	(0.2, 3.7)
Officer vs. E5-E9	13.3	7.4	1.3	(0.5, 3.5)	10.9	1.0	(0.1, 8.0)	7.2	10.6	0.7	(0.3, 1.7)	12.3	0.5	(0.0, 5.4)
**Education**														
H.S.- Alternate Education- GED	19.3	10.9	**2.3***	**(1.0-5.1)**	10.9	2.2	(0.3-15.0)	20.1	17.1	1.1	(0.6-2.2)	8.7	2.2	(0.2-28.5)
High School diploma	56.3	73.0		-	69.0		-	65.9	63.9		-	63.8		-
Some college	4.4	4.6	1.2	(0.3-5.0)	1.5	3.5	(0.0-393.8)	4.3	4.4	1.0	(0.3-3.3)	3.4	1.2	(0.0-65.0)
College or higher	20.0	11.6	**2.2***	**(1.0-4.9)**	18.6	1.3	(0.3-6.5)	9.7	14.6	0.6	(0.3-1.4)	24.1	0.4	(0.1-2.3)
**Age At Entry to Army Service**														
17-20	53.3	59.7		-	55.7		-	60.2	56.1		-	52.3		-
21-24	25.5	23.6	1.2	(0.6-2.4)	30.2	0.9	(0.2-3.6)	29.2	26.3	1.0	(0.6-1.9)	31.0	0.8	(0.2-4.2)
25+	21.3	16.7	1.4	(0.7-3.0)	14.2	1.6	(0.3-9.3)	10.6	17.6	0.6	(0.3-1.2)	16.7	0.5	(0.1-4.3)
**Current age**														
18-24	23.5	34.6	0.5	(0.3-1.2)	35.0	0.6	(0.1-2.5)	34.3	27.9	1.0	(0.5-1.8)	36.8	0.8	(0.1-4.4)
25-29	38.2	30.8		-	31.6		-	36.8	28.4		-	29.7		-
30-34	17.7	14.9	1.0	(0.4-2.2)	12.3	1.2	(0.2-8.9)	16.4	19.8	0.6	(0.3-1.3)	11.0	1.2	(0.1-15.0)
**35+**	20.7	19.7	0.8	(0.4-1.9)	21.0	0.8	(0.1-4.5)	12.5	23.9	**0.4***	**(0.2-0.9)**	22.5	0.4	(0.1-3.4)
**Deployment Status**														
Never Deployed	18.9	21.8	0.8	(0.4,1.7)	22.7	0.8	(0.2,3.5)	28.7	16.6	**2.0***	**(1.1,3.7)**	21.9	1.4	(0.3,8.1)
Previously Deployed	81.1	78.2		-	77.3		-	71.3	83.4		-	78.1		-
**Years of Active Duty**														
1-4	24.6	42.4		-	35.5			32.6	32.7		-	36.3		-
5-8	41.5	26.3	**2.7***	**(1.3, 5.6)**	31.0	1.9	(0.4, 8.8)	39.6	32.0	1.2	(0.7,2.3)	29.9	1.5	(0.3,8.7)
9+	34.0	31.3	1.9	(0.9, 3.9)	33.6	1.5	(0.3, 6.6)	27.8	35.2	0.8	(0.4,1.5)	33.8	0.9	(0.2,5.1)

*p<0.05 (cases vs controls)
Table abbreviated due to space constraints. Results for excluded variables available upon request.

© 2019 Dempsey CL et al. *JAMA Network Open.*
